# Post-traumatic stress disorder symptoms among internally displaced persons: unveiling the impact of the war of Tigray

**DOI:** 10.1007/s44192-024-00069-2

**Published:** 2024-05-28

**Authors:** Aregawi Gebreyesus, Asqual Gebreslassie Gebremariam, Kokob Gebru Kidanu, Solomon Gidey, Hansa Haftu, Afewerki Tesfahunegn Nigusse, Fiyori Shishay, Liya Mamo

**Affiliations:** 1 Department of Epidemiology, College of Health Science, Mekelle University, Mekelle, Tigray Ethiopia; 2 Department of Psychiatry, College of Health Science, Mekelle University, Mekelle, Tigray Ethiopia; 3 School of Medicine, College of Health Science, Mekelle University, Mekelle, Tigray Ethiopia; 4 Department of Biostatistics, College of Health Science, Mekelle University, Mekelle, Tigray Ethiopia

**Keywords:** Post traumatic stress disorder, Internally displaced persons, The War of Tigray

## Abstract

**Introduction:**

Due to the war in Tigray, 2.1 million people (31% of the total population) were internally displaced. Epidemiological evidence shows that the burden of mental health is higher in war/conflict and post-conflict areas of the world compared to non-conflict places, especially for those who have experienced targeted ethnic violence as a result of civil and political unrest. Post-traumatic stress disorder is one of the common psychiatric disorders experienced during war. Thus, this study aimed to assess the level and aggravating factors of PTSD during the war in Tigray.

**Methods:**

A community-based cross-sectional study was conducted among 2132 IDP family heads in Tigray from August 6–30, 2021. Study participants were recruited using a multi-stage sampling technique. Data were collected using a pretested structured questionnaire through face-to-face interviews. The PCL-C checklist, derived from DSM-IV criteria, was used to assess the magnitude of post-traumatic stress disorder. The entered data were exported to the SPSS version 26 statistical package for analysis. Summary statistics were computed, and logistic regression analysis was used to investigate factors associated with developing PTSD.

**Results:**

A total of 2071 IDPs were surveyed with a response rate of 99.7%. The survey revealed that the level of PTSD among community-hosted IDPs was 57.7%; 95% CI 55.5%-59.8%. Older age (> 50) (AOR 3.1, 95% CI 1.497–6.421), primary and secondary school attendance (AOR 2.1, 95% CI 1.344–3.279; and 1.697, 95% CI 1.067–2.7) respectively, internally displaced persons with a family size of > 6 members (AOR 1.821, 95% CI 1.124–2.95), disability due to the war (AOR 1.702, 95% CI 1.077–2.69), and loss of contact with family members (AOR 1.472, 95% CI 1.032–2.099) were significantly associated with PTSD.

**Conclusion:**

The overall level of PTSD among cIDPs was found to be high (57.7%). Almost every other IDP developed this serious mental health syndrome. Immediate psycho-social health intervention is needed by local and international organizations in collaboration with governmental and non-governmental institutions based on the study's findings.

## Introduction

Internally Displaced Peoples (IDPs) are a group of people who have been obliged to leave their permanent residence areas as a result of or to escape/avoid armed conflict or disaster, which may be due to manmade or natural causes but remain within their country. Worldwide, there are more than 100 million individuals involuntarily displaced from their homes due to conflict and insecurity. Among those displaced, 53.2% are IDPs, with the majority of them living in low-income countries [1–6]. Epidemiological evidence shows that the burden of mental health is higher in conflict and post-conflict areas of the world compared to non-conflict places, especially for those who have experienced targeted ethnic violence and civil or political unrest [7]. Displacement itself is a psychologically traumatic process because these individuals may face traumatic events/stressors [8].

The most common psychopathology seen in displaced individuals is post-traumatic stress disorder (PTSD), with prevalence rates varying from 14 to 37% [8–10]. Many patients experience PTSD after exposure to traumatic or stressful events, which is expressed through symptoms of intrusion, avoidance, mood swings, and sleeplessness [7, 9]. Other literature and epidemiological surveys also show that PTSD is among the most common mental health problems in refugees and IDPs, with prevalence rates ranging from 30 to 40% due to the effects of war and post-migration living difficulties [7, 11, 12]. The rates of PTSD in IDPs of Turkey and Syria were reported as 21.4% and 36.9% respectively [7, 8, 11, 13]. A systematic review and meta-analysis in sub-Saharan African countries showed an overall prevalence of PTSD across all studies to be 22%, with a significant difference between war-exposed (30%) and war-non-exposed (8%) populations [10]. Prevalence studies on PTSD in IDPs from different African countries have also been conducted, revealing high rates in Uganda (54%), Nigeria (42.2%), and southern Ethiopia (Gede’o–58.4%). Many studies reported that age, sex, frequency of the traumatic event, type of the event, disability due to traumatic event, anxiety, lack of basic needs after trauma, loss of someone in love, and substance use were significantly associated with PTSD [9, 14–17].

The war of Tigray began on November 4, 2020, between the Tigray regional state and the federal government of Ethiopia, occurring in various locations and borders of Tigray. After the initial conflict, the Ethiopian government declared war with their allies, which consisted of the Ethiopian National Defense Forces (ENDF), Eritrean Defense Forces (EDF), Somali troops, forces from the neighboring region of Amhara (Amhara militia, vigilante group fano), and militias/special forces from other Ethiopian regional states. The war lasted for a full two years with three phases. In the phase-I of the full-scale war persisted for 8 months throughout the entirety of the Tigray region, resulting in the displacement of numerous Tigrayans, who became internally displaced persons (IDPs) within Tigray or sought refuge in neighboring countries like Sudan. According to reports from the UN and USAID, more than 2.1 million people were internally displaced, with over 71,000 seeking refuge in Sudan [18]. Although the majority of Tigray has been liberated after the 8 months of conflict, clashes continue in border areas, and certain parts of the region, such as western Tigray, eastern Tigray, and southwest Tigray, remain under the occupation of Ethiopian forces and their allies [19, 20]. Throughout this war, the civilian population of Tigray has endured immense suffering, being subjected to torture, indiscriminate killings, and widespread humiliation. Many have been forcefully displaced, with their properties seized. Additionally, they have been subjected to assault and forced labor, including acts of sexual slavery, where women and girls have been raped in groups, often in front of their families and loved ones. Soldiers have even inserted foreign objects, such as nails, stones, and sponges, into their genitalia [21–24]. The war has resulted in the displacement of numerous Tigrayans, especially those living in western Tigray, who have been forced to flee to various parts of the region. These displacements have been carried out through violent means, orchestrated by the Amhara Militia and fano, comprised of residents from the neighboring Amhara region. They have conducted house-to-house searches, using firearms, sticks, and knives to massacre civilians and forcefully expel them from their homes. Furthermore, these displaced individuals have been prevented from seeking refuge in neighboring countries such as Sudan. The actions of the Amhara Militia and Fano have included looting, destroying properties, ethnically driven cleansing, and subjecting the affected population to verbal and physical abuse of unimaginable proportions. Mass arrests, killings, and instances of sexual slavery have become distressingly commonplace, witnessed by horrified family members and relatives. Given the persistent military confrontations and the overarching security threats, the majority of those displaced have sought limited refuge in schools, places hosted by the community, or shelters with their extended families [18, 21, 25–35].

The War of Tigray has brought about significant consequences, including the displacement of millions of people, widespread starvation due to the total blockade of humanitarian aid, and the tragic loss of hundreds of thousands of lives [24]. These harsh realities have exposed the community to unimaginable levels of stress, creating the potential for enduring mental health problems that can affect the entire population. However, existing research on the health of internally displaced persons (IDPs) has primarily focused on specific health conditions, leaving us with limited knowledge regarding the overall impact of violent conflict and internal displacement on health outcomes. Moreover, the majority of mental health studies have been confined to IDP camps, leaving a scarcity of data and evidence for those displaced individuals who are hosted within the community. To show the burden of PTSD in Tigray during this war, reaching far more displaced individuals who didn't reside in camps was needed. So along with other studies that were done in IDP camps, this study tried to show the burden of PTSD by also studying community-hosted displaced individuals. Additionally, living in camps in numbers in unsuitable environments can also aggravate the stress level but since this study is among community hosted, it will only show us factors that are related to the displacement alone which are not related to the living conditions in camps and also come up with new factors that are associated with living in a hosting community. Saying this, this study tried to unveil the impact of the war on IDPs and to intervene with immediate psycho-social health support through the collaboration of local and international organizations that are preferably factor-specific based on the study's findings to ensure a healthy community that will support themselves and also participate in the reconstruction of the region in the long run. Hence, this study aimed to assess the level of post-traumatic stress disorder (PTSD) and identify factors associated with its development among IDPs residing with community hosts.

## Methods and materials

### Study setting

This study was conducted in all zones of Tigray except the western zone and some parts of districts in the Eastern zone. These areas were excluded as the areas were under the control of the invading forces. Administratively Tigray was divided into 7 zones and 52 Districts (18 urban and 34 rural). Both urban and rural settings were considered for the study.

### Study design, population, and period

A community-based cross-sectional survey was conducted to collect data on 48 Districts of Tigray in August 06–30/ 2021. This design was preferred because of its feasibility to conduct in areas of armed conflict. All communities hosted internally displaced individuals in the selected zones, districts, and *Tabias* participated in the study.

### Sample size and sampling technique

The sample size was determined for both specific objectives using a single population proportion (i.e. an overall level of PTSD) and a double proportion population formula (i.e. for the second objective). We took the sample size for the second objective based on the following assumptions: prevalence of the exposure was 25%, 18.3% based on a study carried out in Diyarbakir [7], 80% power, 95% level of confidence, 1.5 design effect and 15% non-response. Based on these assumptions the minimum sample size was required 2132.

A multi-stage sampling technique was used. All zones in Tigray were selected except the western zone and then 48 woredas were randomly selected and a systematic random sampling was employed to select HHs from the selected enumeration sites. The list/sampling frame of households that hosted IDPs was taken from the social affairs of the districts and towns. The smallest study unit of this survey was systematically selected households. We interviewed both the family heads of the displaced and host households. When there were 2 or more displaced families in one household, one was selected using simple random sampling. However, if the families came from different areas and if the one displaced is single or with no family and the other displaced is with his/her family, the displaced family (the one displaced with family) was selected purposively.

### Variables

The outcome variable was PTSD (Post Traumatic Stress Disorder): it was measured by summation of the group of symptoms (witness to traumatic life event, intrusive symptoms related to the event, avoidance of stimuli related to the event, negative alteration on cognition and mood associated with the event and alterations on arousal and reactivity related to the event). PTSD was assessed using 17 Likert questions (1–5) questions which indicate the severity of the symptoms. Finally, we declared that participants who scored 44 and above were considered to have PTSD [8, 17, 36, 37].

### Independent variables

#### Socio-demographic characteristics

Age, sex, marital status, educational status, occupation before the war, religion, length of stay after displacement.

#### Economic related variables

Fixed asset before the war, condition of the fixed asset after war, Shortage of necessities, Amount of fixed asset lost estimated by money.

#### Health-related variables

Losing a family member because of the war, Disability status, medical condition and status of a biological relationship with the dead or lost family member, treatment for chronic medical illness.

### Data collection tool, technique, and procedure

The tool is developed from different literatures and it had five parts: (1) socio-demographic data, (2) Economic-related questions (3) health-related questions, (4) Checklist for PTSD based on the criteria of Diagnostic Statistics Manual for Mental Health Disorders (DSM-IV). The PCLis a 17-item self-report measure reflecting DSM-IV symptoms of PTSD. The PCL-C (civilian) asks about symptoms of generic ‘‘stressful experiences’’ and can be used with any population. This version simplifies assessment based on multiple traumas because symptom endorsements are not attributed to a specific event. In many circumstances, it is advisable to also assess traumatic event exposure to ensure that a respondent has experienced at least one event that meets DSM-IV Criterion A. The 17 items offered five response choices ranging from not at all (scores 1 point) to extremely (scores 5 points) with a total score of 85 [8, 17, 36, 37]. It is translated into Tigrinya, the local language, and tested its reliability. The Cronbach’s alpha value obtained in our study varies from 0.926 to 0.929 for each of the 17 questions regarding the checklist obtained from the Diagnostic Statistics Manual for Mental Health Disorders (DSM-IV) [40].

An interviewer-administered structured questionnaire was used to collect the data using trained data collectors and supervisors. The technique of the data collection uses complete and paper-based interviews at the household level by trained data collectors and supervisors. 50 supervisors and 192 data collectors participated in this study. During the data collection, Tigray was at full siege and there was no salary, cash, or bank system, the supervisors and data collectors were not paid. Due to the nature and severity of the war, there were challenges to data collectors and supervisors, including security concerns and potential trauma experienced by interviewers.

### Operational definition

#### Internally displaced persons

Are those who are displaced internally from their residents due to the war in Tigray [4, 38, 39].

#### Community internally displaced people

Individuals who are displaced from their homes because of the war confrontation between the federal government of Ethiopia and the regional government of Tigray and hosted within the community as IDPs.

#### PCL-C

Is a tool used to assess post-traumatic stress disorder among traumatic event survivors. This tool assesses the feeling of the past 30 days. The dichotomous cutting point for this tool is > 44. A person with a total sum score > 44 was considered to have PTSD according to DSM IV, PCL V [8, 36, 40].

#### Disability

That is impairment in physical body functions or structures while activity limitations are difficulties encountered by an individual in executing tasks or actions as a result of the war.

#### Woreda

Is the equivalent name for the district.

#### Tabia

Is the lowest administrative unit in a woreda.

### Data quality assurance and management

The questionnaire was first prepared in English and translated to the local language Tigrinya language and then it was again translated back to English. The three-day training was given to data collectors and supervisors about detail items of the questionnaire and field guide. One week before the actual data collection, a pretest was done from a similar population outside the study area, and based on the findings of the pretest, minor modifications of questions, wordings, phrases, and time required to interview respondents were made. The questionnaire was checked for completeness, missed values, and unlikely responses and then manually cleaned up on such indications. Then data were coded and entered into a computer using Epi-data version 3.7 for entry customizing and skip benefits. After data cleaning, it was exported to SPSS version 26 computer software packages. Data were cross-checked for consistency and accuracy.

### Data analysis

After being transferred to SPSS we cleaned the data and prepared it for analysis. Using SPSS software descriptive, bivariate, and multivariable logistic regression analysis was done. Descriptive statistical analysis such as simple frequencies, percentages, measures of central tendency, and measures of variability were used to describe the characteristics. Then the information was presented using frequencies, summary measures, and tables. Tested chi-square and degree of freedom to look at the separate relationship with the outcome variable. In the bivariate analysis each independent factors of PTSD were taken to see the association and all variables with p value of less than 0.25 were included in the multivariable logistic regression analysis. For all of the analysis, the data was checked for, outliers, multi-collinearity, and reliability of PTSD using VIF and Cronbach’s alpha respectively. For the declaration of the significant factors, the cut point was p-value < 0.05.

### Ethical clearance

Ethical clearance was obtained from Mekelle University, College of Health Science Ethical Review Board on August 2, 2021, with IRB Ref: MU-IRB 1907/2021. Additional support letter was written from the Tigray Bureau of Health, and *Woreda* health offices. The HH heads (participants of the study) were communicated and written consent was obtained before the data collection. To protect the participants, no individual identifying information was collected. Respondents were informed of their right to withdraw from the study at any time with no subsequent harm for refusal of participation. All methods were carried out by relevant guidelines and regulations or declaration of Helsinki.

## Result

### Characteristics of the respondents

A total of 2071 IDPs were asked in this study with a response rate of 99.7% using the PCL-C checklist derived from DSM-IV criteria to know the magnitude of post-traumatic stress disorder. Among them around 1140 (55.3%) were Female and 923(44.7%) male respondents. The median age of the respondents was 31 (IQR 26–40) years and the ages of the majority of the respondents ranged from 21 to 35 years. Most of them around 776 (38.2%) were displaced from Western Tigray, followed by North West and Eastern shared 360 (17.7%) and 344 (17%) of each respectively. Regarding the occupation status before the war, most of the internally displaced persons were merchants accounting for 614(29.8%) followed by 373 (18.1%) government employees and 319 (15.5%) farmers. Currently, most of them around 635 (31.8%), 520 (26.1%), and 338 (17%) are living in the eastern, central, and northwest of Tigray respectively. (Table 1).

**Table 1:** Socio-demographic characteristics of respondents with a magnitude of PTSD, from August 06–30, 2021 (n = 2071)

Variable	Category	PTSD		^χ2^	DF (P-value)	
		Yes %	No %			
Sex	Male	516 (55.9)	407 (44.1)	2.047	1 (0.153)	
	Female	673 (59)	467 (41)			
Age	< 20	55 (42)	76 (58)	29.060	3 (0.000002)	
	21–35	651 (55.8)	515 (44.2)			
	36–50	364 (61.5)	228 (38.5)			
	> 50	114 (70.4)	48 (29.6)			
Marital status	Single	222 (49.6)	226 (50.4)	15.303	3 (0.002)	
	Married	846 (59.7)	572 (40.3)			
	Divorced	77 (61.6)	48 (38.4)			
	Widowed	36 (60)	24 (40)			
Educational Status	No formal education	96 (55.5)	77 (44.5)	11.868	3 (0.008)	
	Primary	375 (64)	211 (36)			
	Secondary	359 (55.1)	293 (44.9)			
	College and above	189 (56.1)	148 (43.9)			
Religion	Christian	1159 (57.5)	857 (42.5)	0.666	1(0.414)	
	Muslim	28 (63.6)	16 (36.4)			
Zone	South	78 (52.3)	71 (47.7)	40.190	(0.0001)	
	Southeastern	49 (46.2)	57 (53.8)			
	Mekelle	34 (34)	66 (66)			
	Eastern	208 (60.5	136 (39.5)			
	Central	104 (53.6)	90 (46.4)			
	Northwest	219 (60.8)	141 (39.2)			
	Western	481 (62)	295 (38)			
Occupation	No job	84 (52.6)	81 (47.4)	13.140	7(0.069)	
	Farmer	206 (64.6)	113 (35.4)			
	Governmental employee	212 (56.8)	161 (43.2)			
	Non-governmental employee	54 (53.5)	47 (46.5)			
	Merchant	361 (58.8)	253 (41.2)			
	Investor	10 (66.7)	5 (33.3)			
	Daily laborer	118 (56.9)	156 (43.1)			
	Other	103 (50.9)	93 (49.1)			
Length time after displacement	< 4 month	132 (57.6)	97 (42.4)	0.117	2 (0.943)	
	4–6 month	139 (56.7)	106 (43.3)			
	> 6 month	917 (57.9)	667 (42.1)			
Displaced family size	1–3	476 (51)	457 (49)	37.62	2 (0.0006)	
	4–6	493 (63.7)	281 (36.3)			
	> 6	122 (68.9)	55 (31.1)			
Property lost	Yes No	971 (59.7) 211 (49.3)	656 (40.3) 217 (50.7)	14.946	1 (0.000111)	
Losing contact with family members	No		925 (54.9)	759 (45.1%)	25.018	1 (0.00005)
	Yes		203 (70.7)	84 (29.3%)		

*DF* degree of freedom

### Prevalence of PTSD and Health-related characteristics

**Fig. 1:**
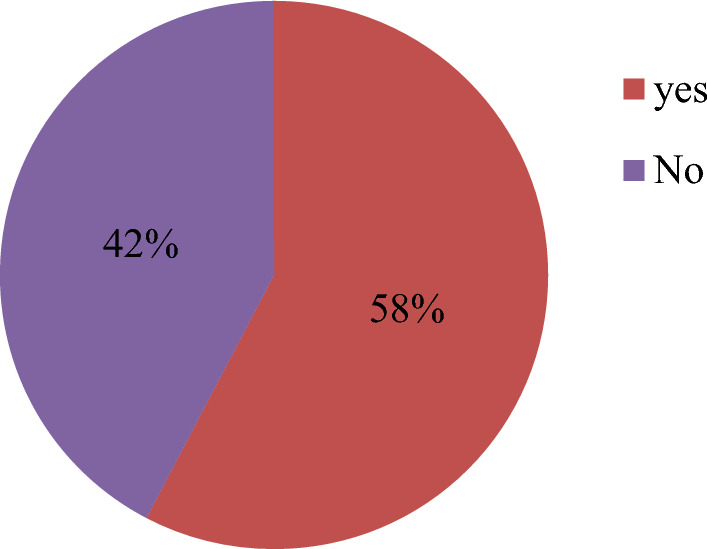
Magnitude of PTSD among internally displaced persons who were hosted in the community in Tigray, 2021

**Fig. 2:**
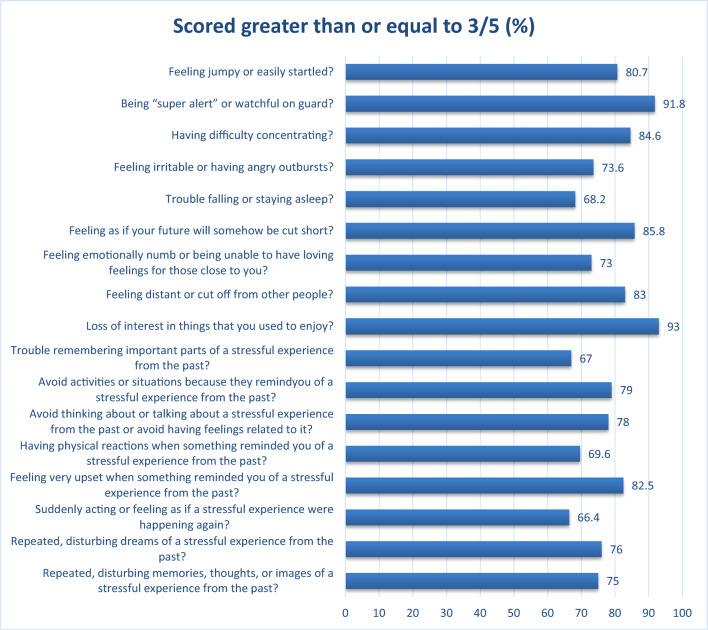
Percentage of PTSD symptoms experienced among IDPs hosted in the community, Tigray, 2021

The prevalence of PTSD was found to be 57.7% 95% CI (55.5%-59.8%) from a total of 2071 individuals who were screened for the PCL-C DSM-IV screening criteria. The level of PTSD did not show a significant difference between categories of sex, religion, occupation, and length of time after displacement with (χ^2^(1) = 2.047, P = 0.153), (χ^2^(1) = 0.666, P = 0.414), (χ^2^(7) = 13.14, P = 0.069) and (χ^2^(2) = 0.117, P = 0.943) respectively. Most of the respondents 717(34.6%) were having moderate PTSD symptoms followed by quite a bit 579 (28%) and little 416 (20%) respectively. Those who had severe PTSD symptoms were about 312 (15.1%). Besides PTSD, around 318 (16%) of the respondents had known medical illnesses and 154 (8%) of them got physical disability because of the war. (Table 2), (Fig. 1), (Fig. 2).

**Table 2:** Health-related characteristics of respondents with a magnitude of PTSD, from August 06–30, 2021(n = 2071)

Variable	Category	PTSD		^χ2^	DF(P-value)
		Yes %	No %		
Dead family member because of the war	No	982 (55)	804 (45)	26.038	1 (0.0000004)
	Yes	147 (73.9)	52 (26.1)		
Faced disability because of the war	No	985 (55.8)	781 (44.2)	11.903	1 (0.001)
	Yes	108 (70.1)	46 (29.9)		
Having a known medical condition	No	925 (56.4)	716 (43.6)	7.155	1 (0.007)
	Yes	205 (64.5)	113 (35.5)

### Factors associated with PTSD

Variables that had a P < 0.25 in the bivariate model were fitted to the final multivariable logistic analysis and participants with age > 50, primary and secondary school level attendants, those who had a family size > 6, those who had disability and who lost contact with their family members were found to be at higher risk of developing PTSD. Respondents with the age of 50 and above were 3.1 times more likely to develop PTSD compared to other age groups (AOR 3.1, 95% CI 1.497–6.421; P < 0.01). Primary and Secondary school attendant respondents were 2.1 and 1.697 more likely to develop PTSD with (AOR 2.1,95% CI 1.344–3.279; P < 0.01 and 1.697, 95%CI1.067–2.7; P < 0.05) respectively. This study also found that those internally displaced persons who have a family size > 6 members and who faced disability because of the war were at 1.821 and 1.702 higher risk of developing PTSD with (AOR 1.821, 95%CI 1.124–2.950; P < 0.05) and (AOR 1.702, 95%CI 1.077–2.690; P < 0.05) respectively. Finally, those individuals who lost contact with their family members were also more likely to develop PTSD (AOR 1.472, 95%CI 1.032–2.099; P < 0.05) respectively. (Table 3).

**Table 3:** Factors associated with PTSD among internally displaced persons in Tigray, from August 6–30, 2021

Variable		PTSD		COR (95%CI)	AOR (95%CI)
		Yes %	No %		
Sex	Male	516 (55.9)	407 (44.1)	1	
	Female	673 (59)	467 (41)	1.137 (0.954–1.355)^*^	1.34 (1.041–1.722)
Age	< 20	55 (42)	76 (58)	1	
	21–35	651 (55.8)	515 (44.2)	1.747 (1.212–2.518)^**^	1.28 (0.788–2.087)
	36–50	364 (61.5)	228 (38.5)	2.206 (1.502–3.240)**	1.548 (0.898–2.668)
	> 50	114 (70.4)	48 (29.6)	3.282 (2.023–5.324)**	3.10 (1.497–6.421)**
Marital status	Single	222 (49.6)	226 (50.4)	1	
	Married	846 (59.7)	572 (40.3)	1.506 (1.216–1.864)**	1.29 (0.965–1.726)
	Divorced	77 (61.6)	48 (38.4)	1.633 (1.089–2.449)*	1.34 (0.784–2.286)
	Widowed	36 (60%)	24 (40%)	1.527 (0.882–2.643)	.937 (0.369–2.381)
Educational Status	No formal education	96 (55.5%)	77 (44.5%)	1	
	Primary	375 (64)	211 (36)	1.426 (1.011–2.011)*	2.10 (1.344–3.279)**
	Secondary	359 (55.1)	293 (44.9)	0.983 (0.701–1.377)	1.697 (1.067–2.700)*
	College and above	189 (56.1)	148 (43.9)	1.024 (0.708–1.481)	1.54 (0.889–2.667)
Zone of origin	South	78 (52.3)	71 (47.7)	1	
	Southeastern	49 (46.2)	57 (53.8)	0.783 (0.475–1.289) *	0.796 (0.426–1.487)
	Mekelle	34 (34)	66 (66)	0.469 (0.278–0.792) *	.631 (0.327–1.218)
	Eastern	208 (60.5)	136 (39.5)	1.392 (0.945–2.051)	1.329 (0.804–2.196)
	Central	104 (53.6)	90 (46.4)	1.052 (0.686–1.613)	1.054 (0.595–1.868)
	Northwest	219(60.8%)	141 (39.2%)	1.414 (0.962–2.078)	1.339 (0.817–2.195)
	Western	481 (62)	295(38)	1.484 (1.043–2.112) *	1.422 (0.904–2.236)
Occupation	Farmer	206 (64.6)	113 (35.4)	1	
	Governmental employee	212 (56.8)	161 (43.2)	0.630 (0.401–0.992)*	1.164 (0.729–1.858)
	Nongovernmental employee	54 (53.5)	47 (46.5)	0.783 (0.591–1.036)*	.708 (0.385–1.301)
	Merchant	361 (58.8)	253 (41.2)	1.097 (0.366–3.288)	1.239 (0.850–1.805)
	Investor	10 (66.7)	5 (33.3%)	0.725 (0.521–1.010)	1.618 (0.290–9.025)
	Daily laborer	118 (56.9)	156 (43.1)	0.569 (0.388–0.833)	1.180 (0.757–1.839)
	No job	84 (52.6)	81 (47.4)	0.569 (0.423–0.873)**	1.036 (0.612–1.754)
	Other	103 (50.9)	93 (49.1)	0.608 (0.423–0.873)**	0.965 (0.595–1.564)
Length time after displacement	< 4 month	132 (57.6)	97 (42.4)	1	
	4–6 month	139 (56.7)	106 (43.3)	0.964 (0.670 − 1.387)	
	> 6 month	917 (57.9)	667 (42.1)	1.010 (0.763–1.337)	
Displaced family size	1–3	47 (51)	45 (49)	1	
	4–6	49 (63.7)	28 (36.3)	1.684 (1.386–1.511)**	1.277 (0.992–1.644)
	> 6	122 (68.9)	55 (31.1)	2.130 (2.047–3.002)**	1.821 (1.124–2.950)*
^a^Property lost	Yes	971 (59.7)	656 (40.3)	0.657 (0.530–0.814)^**^	0.899 (0.664–1.218)
	No	211 (49.3)	217 (50.7)	1	
Dead family member because of the war	No	982 (55)	804 (45)	1	
	Yes	147 (73.9)	52 (26.1)	2.315 (1.664–3.218)**	1.501 (0.977–2.304)
Faced disability because of the war	No	985 (55.8)	781 (44.2)	1	
	Yes	108 (70.1)	46 (29.9)	1.852 (1.302–2.662)**	1.702 (1.077–2.690)*
Losing contact with family members	No	925 (54.9)	759 (45.1)	1	
	Yes	203 (70.7)	84 (29.3)	1.983 (1.511–2.602)**	1.472 (1.032–2.099)*
^b^Having a known medical condition	No	925 (56.4)	716 (43.6)	1	
	Yes	205 (64.5)	113 (35.5)	1.404 (1.094–1.802)**	1.116 (0.808–1.541)

^a^Lost animal, cereals, machinery, house properties

^b^DM, HTN, Epilepsy, heart disease, chronic respiratory diseases

**Significant at P < 0.01, * Significant at P < 0.05

## Discussion

The purpose of this research was to evaluate the extent of post-traumatic stress disorder (PTSD) and determine the factors linked to it. The survey findings indicated that the prevalence of PTSD among internally displaced persons (IDPs) living within the community was 57.7%, with a confidence interval of 95% ranging from 55.5% to 59.8%. Interestingly, this result aligns with a previous study conducted in southern Ethiopia, which reported a PTSD prevalence of 58.4% [8]. This similarity could be attributed to the geographical proximity and shared socio-cultural and economic backgrounds of the participants in both regions.

However, the results of this study showed a higher prevalence of PTSD compared to findings from Turkey (21.4%), Uganda (54%), and Syria (36.9%) [8, 11, 17]. Several factors could explain this difference. Firstly, there may be variations in the socio-demographic characteristics of the participants. Additionally, the sample size in Turkey was smaller, consisting of only 433 participants. Furthermore, different assessment tools were used in each study, with Turkey utilizing the Traumatic Stress Symptom Scale, Uganda using the Harvard Trauma Questionnaire, and Syria employing the Screen for Posttraumatic Stress Symptoms [3, 12, 14].

Moreover, the severity and intensity of traumatic events experienced in Tigray, such as atrocities and gang rape by military forces, were exceptionally severe and distressing. In contrast, the availability of support from various organizations in Turkey and Syria may have contributed to lower PTSD rates in those regions. It is important to note that Tigray has been under siege for an extended period, resulting in limited access to medication and food, particularly within IDP camps. Unfortunately, this lack of resources extends to mental health and psychosocial support, further prolonging the suffering of individuals with PTSD.

The results of this study showed a lower prevalence of post-traumatic stress disorder (PTSD) compared to findings from Iraq (60%) and southwestern Uganda (67%) [13, 17]. The difference in prevalence could be attributed to variations in sample sizes, with Iraq having 988 participants, Uganda having 387 participants, and the current study in Tigray having 2071 participants. Additionally, the use of different assessment tools may have contributed to the disparity in results. The study in Iraq utilized the PTSD Checklist for DSM-5, while the study in Uganda employed the MINI International Neuropsychiatric Interview (MINI), whereas the current study utilized the PCL-C 17-item scale [13, 15, 40].

Individuals aged 50 and above were found to be 3.1 times more susceptible to developing post-traumatic stress disorder (PTSD) compared to other age groups. This finding is consistent with a study conducted in northern Uganda [14]. The possible explanation for this could be that older individuals have witnessed the death of their children, and the rape of their daughters in their presence, and have experienced physical and emotional trauma inflicted by military forces during a stage in life when they should be respected. Moreover, older people may have been exposed to previous traumatic events related to war, which can be triggered by similar events occurring in the current conflict in Tigray [22–24]. Additionally, they may struggle to adapt to the new lifestyle in internally displaced persons (IDPs) camps, as it can be challenging to rely on others for their basic needs after having worked their entire lives to be self-sufficient. The presence of chronic medical conditions and dietary restrictions that cannot be met further contribute to the development of PTSD as additional stressors.

Furthermore, this study revealed that IDPs who experienced disabilities as a result of the war were at a 1.702 times higher risk of developing PTSD. This association can be attributed to the fact that becoming disabled is a profoundly traumatic experience that often leads to PTSD. When individuals become disabled suddenly, without access to medical services, financial support, legal aid, and social assistance, it becomes difficult for them to cope with the incident. In Tigray, during the ongoing conflict, many people have been exposed to incidents that have resulted in disabilities, providing evidence to support this association [2, 41–43].

The likelihood of developing PTSD is 2.1 and 1.697 higher for individuals who attended primary and secondary school compared to those without formal training. This could be because educated individuals are often engaged in productive activities within the community, and losing these activities and financial stability can be a significant source of stress, especially for breadwinners. Additionally, in IDP centers, these individuals continue to take on roles as organizers and community members, which exposes them to secondary trauma when working with people who have experienced traumatic events and share their stories [5, 43–45].

Furthermore, this study found that internally displaced persons with a family size larger than six members were at a 1.821 higher risk of developing PTSD. In the Tigray crisis, the daily struggle for food and survival is a major concern for everyone. Parents with larger families are particularly worried about how to provide for their loved ones, as there is a scarcity of food, medication, clean water, and other essentials [36, 43]. If a person already has a psychological disturbance like an anxiety disorder and then experiences a traumatic event, the risk of developing PTSD becomes even higher. Therefore, the coexistence of another psychological disturbance may increase the vulnerability to PTSD among parents with larger families who constantly worry about their family's survival [20, 35].

Additionally, individuals who have lost contact with their family members are also more likely to develop PTSD. This finding aligns with research conducted in Croatia on internally displaced people. While not everyone who experiences a traumatic event develops PTSD, the risk is higher when multiple traumatic events occur simultaneously. In the Tigray war, almost everyone witnessed traumatic events [23, 24]. So those who lost contact with their families and experienced these events are at a heightened risk of developing PTSD. Social support is one protective factor in times of crises for people to cope. When there is separation from family members there is disconnection and it leads to psychological distress [21, 22, 27, 46]. However, when there is separation from family members, it leads to disconnection and psychological distress. In Tigray, where access to phones, internet, transportation, and banking services was limited, it became difficult to know the whereabouts and status of family members. This added stressor of worrying about their well-being could be associated with the development of PTSD [21, 28].

## Strengths and limitations of the study

This study was conducted in all woredas/districts of Tigray (with a large sample size) at the community level, which may have external validity and generalizability for internal and other IDPs globally. However, there are limitations to this study. Since it was cross-sectional, it did not establish a temporal causal relationship between the outcome variable and the significant factors. Additionally, the study focused only on interviewing the HH heads of the cIDPs, excluding other members, which may have underestimated the findings. Individuals with more severe mental health issues might be less likely to participate in face-to-face interviews. The data collection took place in August 2021, while the war and siege continued afterward. This might not fully capture the evolving nature of the conflict in Tigray and could underestimate the level of PTSD in the region, providing an incomplete picture. There could have been significant changes in the prevalence of PTSD and associated factors after the data collection period. In addition to that, the PCL-C in itself is not diagnostic and is a self-report tool to identify whether a person needs further assessment for diagnosis and treatment of PTSD by qualified professionals.

## Conclusion

The overall level of PTSD among cIDPs was found to be high (57.7%). This has been characterized by unacceptably high levels of mental health problems and every other IDP has developed this very serious mental health syndrome. This needs immediate psycho-social health intervention by local and international organizations with the collaboration of governmental and non-governmental institutions based on the study's findings. Further investigation on resilience amongst persons exposed to trauma and not exhibiting signs of PTSD is also required. Those IDPs displaced due to the war of Tigray still returned to their home after a one-year peace agreement between the federal government of Ethiopia and the Tigray state government. Applying the peace agreement not only enables the internally displaced persons (IDPs) to return to their homes but also supports their healing process and assists them in rebuilding their lives.

## Data Availability

All the data supporting the findings is contained within the manuscript, when there is in need the data set used for the present study's conclusion can be accessible from the corresponding author upon reasonable request.

## Abbreviations

AOR: Adjusted odd ratio
COR: Crude odd ratio
DSM: Diagnostic statistics manual for mental health disorders
cIDPs: Community hosted Internally displaced peoples
PTSD: Post traumatic stress disorder
PCL-C: Post‐traumatic stress disorder checklist: Civilian
SPSS: Statistical package for social science
DSM: Diagnostic statistics manual for mental health disorders
